# Nitrogen output in the urban environment using a vegetarian canine diet

**DOI:** 10.1371/journal.pone.0257364

**Published:** 2021-09-23

**Authors:** Lena Ingenpaß, Amr Abd El-Wahab, Cristina Ullrich, Mareike Kölln, Marwa F. E. Ahmed, Christian Visscher, Josef Kamphues

**Affiliations:** 1 Institute for Animal Nutrition, University of Veterinary Medicine Hannover, Foundation, Hannover, Germany; 2 Department of Nutrition and Nutritional Deficiency Diseases, Faculty of Veterinary Medicine, Mansoura University, Mansoura, Egypt; 3 Department of Hygiene and Zoonoses, Faculty of Veterinary Medicine, Mansoura University, Mansoura, Egypt; University of Lincoln, UNITED KINGDOM

## Abstract

Pet owners are increasingly concerned about the links between health status, animal welfare, environmental impacts, climate change and consumption of animal products. Accordingly, many owners are increasingly interested in vegetarian diets for themselves and their companion animals. However, such diets should be investigated nutritionally regards digestibility as well as on fecal quality and nitrogen output. In light of this trend, six Beagle dogs were included in a cross-over experimental design and offered a vegetarian diet containing wheat gluten (8.81%), rice protein (8.81%) and sunflower oil (6.84%) or an meat-based diet containing poultry meal (19.5%) and poultry fat (5.23%). The dogs received extruded complete diets for 12 days (adaptation and collection period, each 6 days). The dogs fed both diets showed a high and identical palatability (scoring of food intake) of the experimental diets. No significant differences occurred regarding digestibility of organic matter, crude protein and crude fat between vegetarian and meat-based diets. However, dogs fed the meat-based diet had higher (p < 0.05) nitrogen-free extract digestibility (89.5%) compared to those fed the vegetarian diet (88.6%). The amount of nitrogen excreted in feces (g)/kg BW^0.75^ was slightly, but not significantly, higher for dogs fed the vegetarian diet compared to those fed the meat-based diet (0.88 vs 0.79). The fecal consistency scores were considered to be within an acceptable range (well formed and firm). The mass of the feces between both groups were similar (62.9 g wet feces/100 g dry matter food) for vegetarian and meat-based diets. Additionally, the fecal dry matter content was comparable between both groups (29.0% and 29.6% for vegetarian and meat-based diets, respectively). In conclusion, the results of this study appear to indicate that virtually the only significant difference between the two diets was lower nitrogen-free extract digestibility in the vegetarian diet. However, the vegetarian diet did not result in a significant difference in amount of nitrogen excreted in feces.

## Introduction

Vegetarians may be defined as persons consuming plant foods, with or without dairy products, eggs and/or honey (i.e., meat only is excluded) as stated by the International Vegetarian Union [1]. The dietary habits of humans have unmistakably an effect on the care of dogs as “family-members” [2]. Given the increasing number of pet owners worldwide who are interested in vegetarian diets for themselves, it is not surprising that they also consider the use of a vegetarian diet for their pets [2, 3]. To put this development into perspective, in the USA alone, with a population of about 330 million in 2021 [4] approximately 56.0% of whom are pet owners, there may be around 20 million vegetarian pet owners [4].

Increased demand for meat is being driven by population expansion and urbanization, which is driving up global supply and consumption [5, 6]. The United Nations’ Food and Agriculture Organization (FAO) forecasted that world meat demand will reach 455 million metric tons by 2050 in 2012 (a 76.0% increase from 2005) [7]. This increased demand is problematic because present large-scale animal husbandry methods are linked to public health issues, environmental degradation, and concerns about animal welfare. With regard to livestock production, agriculture contributes to environmental concerns such as greenhouse gas emissions, land use and water use [6]. Climate change is rapidly developing into the greatest environmental issue for today’s and subsequent generations. Animal agriculture’s industrialisation is a major contribution to worldwide environmental degradation and climate change [8]. In a 2018 report, the United Nations Intergovernmental Panel on Climate Change stated that greenhouse gas emissions must be decreased by 45.0% by 2030 [9]. Lastly, with regard to animal welfare concerns, each year approximately 80 billion of animals are slaughtered and about more than 155 million tonnes of seafood are produced in relation to human food systems [10]. Based on several previous studies, Rubio et al. [11] stated that the raw material inputs (i.e., animals) for conventional meat production are fundamentally unsanitary, inefficient, and sentient. Several externalities could be avoided by removing meat from the manufacturing process [11]. As livestock farming has become more large-scale, as has pet animal keeping, there has been a greater difference in fecal nutrient distribution patterns, resulting in increased localized nitrogen (N) burdens to soil and raising concerns about the environmental impact of these activities [12]. Nitrogen has many effects because it exists in various reactive forms in environmental media (e.g., air, water, and soil) [13]. While nitrate mobilized by leaching and run-off damages water quality, gaseous N compounds can have severe impacts on air quality and climate change. Due to insufficient application of feces/manure, for example, the latter causes significant nitrogen accumulation in soils and rivers (eutrophication), posing a serious environmental concern [14]. To date, only local and regional efforts have been made to minimize N pollution, such as regulating nitrate concentrations in groundwater or limiting nitric oxide emissions to urban airsheds. But also locally in urban areas, the input of N into the environment can be relevant for sensitive habitats if the feces are not removed.

Overall, the motivations reported for switching to non-animal products include well-being concerns as well as sustainability/environmental hazards [6, 7]. Traditionally, commercial pet foods were obtained mostly from the human food industry as animal and plant by-products, which has been considered a highly sustainable process [15]. Since dogs display many characteristics of an omnivore, they are not generally described as purely carnivorous species anymore [16, 17]. Thus, the behavioral and physiological adaptations to a high dietary diversity–including plant feed–became necessary [18]. Accordingly, interest in ‘alternative’ diets, including vegetarian diets, is likely to grow [4]. However, these diets must be nutritionally complete and reasonably balanced. The proper formulation of vegetarian food for dogs is a challenge. Therefore, almost half of pet owners interested in providing vegetarian diets seek advice to ensure the nutritional adequacy of these products, as there are different vegetarian diets available on the pet food market [4, 19].

Protein is an essential component of dog diets, providing amino acids for the various physiological functions [20]. The use of highly digestible protein sources results in a reduced amount of protein entrance in the large intestine [21]. Driven by increasing concerns about health, the welfare of agricultural animals, and the environment, pet owners are increasingly interested in diets and lifestyles which include fewer meat products [2]. Along these lines, screening new protein sources that exist in nature, which may emulate meat without the environmental and animal welfare impacts associated with animal farming may be an approach that appeals to a wider pool of pet owners. Therefore, the objective of this study was to compare the apparent nutrient digestibility of a meat-based control diet (poultry meal) and a vegetarian diet (wheat gluten and rice protein) with a similar dietary carbohydrates origin. Moreover, this study aimed to test the effect of these both diets on the fecal nitrogen output and the fecal characteristics.

## Materials and methods

This study protocol was reviewed and approved by the Animal Welfare Officer of the University of Veterinary Medicine Hannover, Foundation, Germany Committee in accordance with the German protocol § 7 of the Animal Protection Law prior to conducting this study.

### Experimental design

Six healthy, unneutered female Beagle dogs (*n* = 6), sourced from University of Veterinary Medicine Hannover, Foundation, Germany, were participated in the digestibility study, with an average body weight (BW) of 9.64 ± 0.68 kg and a median age of three years. The body condition score during the whole experimental trial varied at 4.98 ± 0.31 of a total of 9 in accordance with Laflamme [22]. The health status of the dogs was checked before the beginning of the experiment by clinical examination and dogs were dewormed, and with up to date vaccinations. The dogs were kept in 3.35 x 2.80 m kennels with daily access to an outdoor playground for exercise and socialization, where they were acclimatized to the tested foods. During the digestibility tests, dogs were housed individually in 4.00 × 2.05 m kennels to enable complete fecal collection. The trial was conducted using a cross over experimental design, in which the six dogs were divided into two groups of three dogs each. Thereafter, the three dogs in each group were shifted.

### Diet and feeding

Two extruded isonutrient diets were produced to meet the adequate requirements for the maintenance of adult dogs [23]. Basically, the extruded diets (MERA Tiernahrung GmbH, Kevelaer, Germany) contained: wheat, broken rice, linseed, sugar beet pulp, brewer’s yeast, palatability enhancer, dicalcium phosphate. The meat-based diet contained (as fresh basis) poultry meal (19.5%) and poultry fat (5.23%) as the main sources of protein and fat. Poultry meal generally contains only ground, rendered, clean parts of the carcass of slaughtered poultry. In contrast, the vegetarian diet contained (as fresh basis) wheat gluten (8.81%) and rice protein (8.81%) with sunflower oil (6.84%) as the main sources of protein and fat. The diet contains vitamin D3 (0.045 g/kg Diet) as the only animal product. It was derived from sheep’s wool, therefore the food is declared as vegetarian and not vegan. Both diets were produced by identical food technologies (temperature: 125–130°C). The ingredients composition of the vegetarian and meat-based foods are presented in Table 1. During this crossover study, each dog was assigned once to a vegetarian diet and once to a meat-based diet. The treatments were balanced according to the animals’ BW (0.40 MJ metabolizable energy/kg BW^0.75^/day). The dogs were in the trial for a total of 24 days (each diet: 6 days adaptation + 6 days fecal collection). The adaptation phase was at least 6 days and allowed the dogs to become acclimated to the test food. The collection phase of 6 days was used for total fecal collection and allowed to collect enough fresh fecal mass for estimating the apparent digestibility and fecal score.

**Table 1:** Ingredient composition of the meat-based diet and vegetarian diet (% as fresh basis).

Ingredient	Meat-based diet	Vegetarian diet
Wheat	30.6	29.1
Broken rice	30.6	29.1
Poultry meal	19.5	-
Poultry fat	5.23	-
Wheat gluten	-	8.81
Rice protein	-	8.81
Sunflower oil	-	6.84
Palatability enhancer	3.00	3.00
Sugar beet pulp	2.94	3.00
Brewer´s yeast	2.00	2.00
Linseed	2.00	2.00
Dicalcium phosphate	1.01	3.24
Minor components	3.12	4.10

### Laboratory analyses

Determination of nutrients in diet and fecal samples was performed in accordance with VDLUFA (Verband Deutscher Landwirtschaftlicher Untersuchungs- und Forschungsanstalten) (Association of German Agricultural Inspection and Research Institutes) methods [24]. The formulated diets were analyzed for dry matter (DM), crude ash, crude protein, crude fat and crude fiber before formulation. The DM content was calculated by drying to a constant weight at 103°C. To determine the crude ash, a part of the sample was incinerated for seven hours at 600°C in the muffle furnace. The crude protein content was calculated after analyzing the total nitrogen content, using the DUMAS combustion method, a catalytic tube combustion method in the elemental analyzer (Vario Max CNS, Elementar Analysensysteme GmbH, Langenfeld, Germany). After an acid digestion in the Soxhlet apparatus, the crude fat content was measured. The content of crude fiber was estimated after washing in diluted acidic and alkaline solutions and subsequent drying at 103°C. Starch content was measured enzymatically, while the sugar content was analysed in accordance with the principles of the Luff-Schoorl method by titration with sodium thiosulfate [24]. After microwave incineration (Ethos lab, MLS GmbH Leutkirch, Germany), the calcium content was determined by atomic absorption spectrometry (Solaar AA Spectrometer M Series, Thermo Fisher Scientific Inc., Waltham, MA, USA) in accordance with AOAC [25]. A photometric characterization of the phosphorus content was based on the vanadate molybdate method in accordance with Gericke and Kurmies [26]. The content of the organic matter and the nitrogen free extract were calculated. Metabolizable energy (ME) contents of the diets were estimated based on their chemical composition, in accordance with the Kamphues et al. [27]. In conformity with Zahn [28], the spontaneous acceptance “food intake scoring”(palatability and the speed of food intake) was divided into three grades (1 = lowest acceptance; 2 = moderate acceptance; 3 = highest acceptance).

### Composition of diets

The moisture content of the diets (as indicated by dry matter in fresh matter) was almost similar (range: 913–941 g/kg) as shown in Table 2. The crude ash level was slightly higher in the meat-based diet (62.4 g/kg DM) than in the vegetarian diet (55.1 g/kg DM). The crude protein content was a little bit higher in the meat-based diet (240 g/kg DM) compared to the vegetarian diet (222 g/kg DM). Also, the crude fiber content was somewhat higher in the meat-based diet (18.1 g/kg DM) than in the vegetarian diet (16.5 g/kg DM). A minor difference was found in the ME content between both diets, where the meat-based diet had about 385 kcal/100 g as fed vs. 368 kcal/100 g as fed for the vegetarian diet.

**Table 2:** Chemical composition of meat-based diet and vegetarian diet.

Parameter	Unit	Meat-based diet	Vegetarian diet
Dry matter in fresh matter	g/kg	941	913
Crude ash	g/kg DM	62.4	55.1
Crude protein		240	222
Crude fat		126	106
Crude fiber		18.1	16.5
Nitrogen free extract		554	600
Metabolizable energy^1^	kcal/100 g as fed	385	368
Calcium	g/kg DM	11.6	9.43
Phosphorus		8.09	8.21

^1^ME content of the diets was estimated in accordance with Kamphues et al. [27].

### Apparent digestibility

The total fecal collection method was used to estimate the rate of nutrient apparent digestibility [29], consisting of an initial phase of 6 days of adaptation to the diet, followed by 6 days of fecal collection. The animals were fed once per day and received water *ad libitum*. The amount of food offered was recorded at each meal and was calculated by formula according to their energy requirements: 0.40 MJ ME/kg BW^0.75^/day, based on the energy requirement prediction equation for maintenance of adult dogs [27]. The food offered was adjusted weekly to keep the animals’ BW constant. Generally, the amount of vegetarian diet offered to the dogs varied between 146 and 186 g/dog as fresh basis, while the amount of meat-based diet offered to the dogs ranged from 140 to 164 g/dog as fresh basis. At the end of each day of collection phase, the collected feces were thawed, mixed and homogenized to receive an individual daily fecal sample. In a pooled sample of 10% of the fresh feces per animal and day, the DM content was determined on each day of the collection phase. The remaining 90% of the sample was frozen at -20°C. By mixing the daily fecal pool samples of an animal into one total pool sample at the end of each collection phase, it was finally possible to create a representative subsample of each animal. The apparent digestibility was determined using this formula: Apparent digestibility (%) = ((food-excreta)/food) x 100 [27].

### Nitrogen estimation and fecal quality

Briefly, to calculate the N-output in the feces, the data of daily energy intake for kg BW^0.75^ (0.95 kcal) and the level of protein for 1000 kcal ME (52.1 g) from previous publications were used according to European Pet Food Industry Federation (FEDIAF) [30]. Thereafter, the following equations were performed to estimate the N-output in the feces:

- Energy requirement for 10 kg BW^0.75^ (kcal) = 10^0.75^×95
- Protein intake for 10 kg BW^0.75^ (g) = (Energy requirement for 10 kg BW^0.75^/1000)×52.1
- N-intake for 10 kg BW^0.75^ (g) = Protein intake for 10 kg BW^0.75^/6.25
- N-output for 10 kg BW^0.75^(g) = N-intake for 10 kg BW^0.75^ –(N-intake for 10 kg BW^0.75^×(apparent protein digestibility/100))

We used the BW of 10 kg in the calculation for N-output in feces according to the average BW of dogs in the current study. Finally, the data of apparent protein digestibility in the current study were combined with standard data from the FEDIAF [30] to calculate the N-output in the feces.

The number of defecations was recorded every day. In accordance with Moxham [31], fecal scores were recorded using a 5-point scale (1 = very hard; 2 = solid, well formed “optimum”; 3 = soft, still formed; 4 = pasty, slushy and 5 = watery diarrhea). The feces shaping scores were determined in accordance with Zieger [32], using a 4-point scale (1 = individual feces mass; 2 = shaped, with strong constrictions at the fecal surface “optimum”; 3 = shaped, with fissures at the fecal surface; 4 = pasty, slushy and 5 = shapeless). Fecal pH was determined by digital pH meter (InLab^®^ Expert Pro, Mettler-Toledo International Inc., Columbus, OH, USA) in a solution of feces and distilled water (1 g/4 mL).

### Statistical analysis

The statistical analysis was performed using the Statistical Analysis System for Windows, SAS^®^ Enterprise Guide^®^, version 9.3 (SAS Institute Inc., Cary, NC, USA). For all parameters, mean values as well as the standard deviation of the mean were calculated. All measured or recorded parameters were analyzed individually and were the basis of the calculation. A Student T-test was carried out, as the assumption of normal distribution could not be rejected. For the statistical evaluation of the feces mass (g/100 g DM diet), a single factor variance analysis with repeated measurements and the Tukey´s test were performed. The significance level was determined at p < 0.05.

## Results

### Apparent digestibility

Food intake was similar at both dietary treatments and no refusals occurred throughout the duration of the feeding trial. The BW of the dogs did not change during the study. The apparent digestibility in dogs fed the experimental diets is shown in Table 3. No significant differences were observed for apparent digestibility of organic matter, crude protein and crude fat between both groups (range: 85.2–86.3%, 80.3–82.3% and 93.5–94.0%, respectively). Nitrogen-free extract digestibility was lower (p < 0.05) for dogs fed the vegetarian diet (88.6%) compared to those fed the meat-based diet (89.5%).

**Table 3:** Apparent nutrient digestibility (%) of nutrients in dogs fed the meat-based diet and vegetarian diet (mean ± SD).

Parameter	Meat-based diet	Vegetarian diet
Organic matter	86.3 ± 1.22	85.2 ± 0.79
Crude protein	82.3 ± 2.83	80.3 ± 2.71
Crude fat	94.0 ± 0.32	93.5 ± 1.05
Nitrogen-free extract	89.5^a^ ± 1.04	88.6^b^ ± 0.60

^a,b^ Means in a row with different superscripts differ significantly (p < 0.05).

### Fecal nitrogen

The amount of N excreted in the feces of dogs fed meat-based diet and vegetarian diet is presented in Table 4. The FEDIAF [30] was used as an official guidelines as well as the estimated crude protein digestibility in the current study to calculate the amount of N excreted in the feces. It was observed that the amount of N excreted in feces (g)/kg BW^0.75^ was slightly, but not significantly, higher for dogs fed the vegetarian diet compared to those fed the meat-based diet.

**Table 4:** Amount of N excretion in feces of dogs fed experimental diets according to FEDIAF [30].

Parameter	Value	
Daily energy intake/kg BW^0.75^	95.0	
Protein level/1000 kcal	52.1	
Energy requirement/10 kg BW^0.75^	534	
Protein intake/10 kg BW^0.75^	27.8	
N intake/10 kg BW^0.75^	4.45	
	**Meat-based diet**	**Vegetarian diet**
Crude protein digestibility (%)	82.3	80.3
Amount of N in feces (g)/kg BW^0.75^	0.79	0.88

### Fecal quality

Defecation frequency was almost similar for both the diets (average 2.30 and 2.57 for meat-based diet and vegetarian diet, respectively) (Table 5). The scores for fecal consistency and shaping were very close to the desired optimum score (score 2) in both groups (Table 5). In the present study, the mass of wet feces was not significantly affected by the diet type (62.9 g wet feces/100 g food on a DM basis). In addition, the fecal DM content followed the same trend as wet fecal output (range: 29.0–29.6%). Also, the difference in the fecal pH value between the start and end of collection periods was comparable for both the groups.

**Table 5:** Fecal characteristics of dogs fed experimental diets (mean ± SD).

Parameter	Meat-based diet	Vegetarian diet
Defecation frequency (n/day)	2.30 ± 0.71	2.57 ± 0.55
Score feces consistency	2.14 ± 0.19	2.08 ± 0.08
Score feces shaping	2.17 ± 0.18	2.11 ± 0.10
Mass of the feces (g wet feces/100 g food on a DM basis)	62.9 ±12.1	62.9 ±1.75
Mass of the feces (g DM feces/100 g food on DM basis)	18.6 ± 3.04	18.9 ± 2.86
DM content of fresh feces (%)	29.6 ± 2.26	29.0 ± 1.75
Fecal pH values during collection		
• Start	6.81 ± 0.52	6.54 ± 0.48
• End	6.64 ± 0.22	6.31± 0.37

## Discussion

During the present study, the effects of protein sources in canine diets were investigated on apparent nutrient digestibility and fecal characteristics. Several factors may affect the digestibility of a diet, including the ingredient source and its chemical composition. In our study, the organic matter digestibility was comparable for vegetarian diet and meat-based diets. However, Bednar et al. [33] stated that the organic matter digestibility was lower for the plant protein-based (soybean meal) diet compared to the poultry meal-based diet. This diet in a study by Bednar et al. [33] contained a higher level of dietary fiber, which could possibly reduce nutrient digestibility. Increasing dietary fiber is, in general, associated with reduced organic matter digestibility in pets [34]. In a recent study, it was found that dogs showed identical acceptance with a comparable organic matter digestibility when offered a vegetarian diet or a vegetarian diet supplemented with feather meal and corn meal or rye or fermented rye [35].

Generally, the crude protein content in both diets met the requirements of adult dogs. Regardless of the protein source, the Association of American Feed Control Officials (AAFCO) recommends a total protein content of 18.0% [29] as the minimal required protein amount in adult dog maintenance food. However, there is no recommendation for the percent of protein, which should come from plants or animals [36]. Although protein adequacy requires the correct amino acids to be absorbed in their appropriate concentrations, the protein digestibility is a valuable indicator of protein nutrition [37]. In the current study, crude protein digestibility was similar for the meat-based diet and vegetarian diet. A significant observation from the data in our study is that the diet tested here had protein digestibility comparable to the normal digestibility (80.0%) described by FEDIAF [30]. Also, the crude protein digestibility of the experimental diets was similar to the values reported by Huber et al. [38] and Bednar et al. [33]. Poultry meal is a common protein source in commercial pet food and its protein digestibility varies considerably, ranging from 77.0% to 90.0% depending on the production process [39, 40]. From another point of view, wheat gluten is a highly digestible protein source with an apparent digestibility of 93.8% [41]. Similarly, inclusion of rice protein concentrate, corn gluten meal, soybean meal, and soybean protein isolate did not negatively influence protein digestibility compared to animal proteins in the canine diets [37]. Nevertheless, Bednar et al. [33] found that the crude protein digestibility of the plant-based diet (containing soybean meal) was significantly lower (82.7%) compared to dogs fed poultry meal-based diet (87.5%). Furthermore, protein digestibility has been repeatedly shown to be influenced by dietary fiber [37]. Generally, there is a controversial debate regarding the effects of fiber on protein digestion in pets. The fermentable carbohydrates may influence protein digestibility through total gastro-intestinal tract metabolism by the microbiome, which can both trap nitrogen as bacterial protein or liberate nitrogen as ammonia [37]. Thus, in our study, it could be that the low dietary crude fiber content for both diets was not high enough to have an effect on the apparent digestibility of organic matter.

In the present study, the type of offered diet did not affect apparent fat digestibility. Our results were comparable to those found by Bednar et al. [33]. The authors in the previous study observed that fat digestibility for dog food composed of poultry by-product meal or soybean meal was similar (90.5% and 88.4%, respectively). In the current study, the dietary fat content was in the range of 106–126 g/kg DM and the apparent digestibility of fat was about 93.5–94.0%. Previous studies [42, 43] observed a similar range of fat digestibility in dogs fed a mixed diet (88.0%-95.0%). Zuo et al. [39] found that the fat digestibility increased to about 97.0% when the amount of dietary fat increased. Also, Hill et al. [44] noted that the digestibility of fat reached about 99.0% when the dogs were offered diets containing a high amount of fat (about 320 g/kg DM). Recently, Abd El-Wahab et al. [35] observed that the digestibility of fat was about 88.0% when dogs fed a diet containing fat of about 72.7 g/kg DM. Nevertheless, Merritt et al. [45] found that the digestibility of fat was about (97.0%) in a meat-based diet with a fat content of about 8.8% as is basis.

The significantly lower nitrogen-free extract digestibility of the vegetarian diet compared to the non-vegetarian diets may be due to the different nitrogen-free extract composition of the protein sources that have been added to the diets (poultry meal vs. wheat gluten, rice protein). In the current study, the nitrogen-free extract content for poultry meal, wheat gluten and rice protein (as raw ingredients) was not identical (10.0, 65.0 and 85.0 g/kg as fresh basis, respectively). In contrast to our data, Urrego et al. [46] found that nitrogen-free extract apparent digestibility was similar for dogs offered poultry meal or wheat gluten-based diets (91.7% and 92.1%, respectively).

Dietary choices have considerable impacts on environmental sustainability [47]. In our study, no differences were found between both diets regarding the N-output by dogs. Compared to a vegetarian diet, a meat-based diet requires more energy, land and water and has greater environmental consequences in terms of erosion, pesticides, waste and greenhouse gas production [48]. For example, in the US, there are more than 163 million pets that consume animal products and therefore the pets food are potentially considered as environmental impacts, including greenhouse gas emission and fecal production [48]. Thus, using vegetarian foods for pets deserve special attention as it could be a tool to decrease the environmental impacts.

In our study, the frequency of defecation between the groups fed meat-based diet or vegetarian diet were not different. Several other studies have reported that soluble or insoluble dietary fiber increases the frequency of defecation [49, 50]. Bednar et al. [33] noted that dogs consuming a plant protein source (soybean meal) diet had higher fecal output compared to those fed poultry meal diet, reflecting higher total dietary fiber content of plant protein sources. Unfortunately, in our study, the soluble or insoluble dietary fiber was not analyzed. Furthermore, Zieger [32] observed that high defecation frequency (3.20/day) was found when dogs were fed a diet with a high crude ash content (141 g/kg DM). However, in the present study, the dietary crude ash content of both diets differed slightly but the frequency of defecation was the same. Similarly, Abd El-Wahab et al. [35] stated that the dietary crude ash content (range: 41.3–53.8 g/kg DM) had no effect on the frequency of defecation/day (range: 1.86–2.29) in Beagle dogs. Thus, the dietary crude ash content might have no influence on the defecation frequency.

In the present study, the fecal consistency scoring was different among dogs fed the different diets. However, it was generally about 2.1 (close to the optimal score value of 2). According to our findings, the non-relevant change in fecal consistency when using a non-vegetarian or a vegetarian compound feed are identical with previous studies by Zentek [51] and Nery et al. [21, 47]. Using soy protein or corn gluten in compound feeds resulted in a positive effect (firming impact) on the fecal consistency [44]. The plant-based diet (soybean meal) resulted in an about 50.0% higher wet fecal output and highest fecal scores compared to poultry meal diets, indicating a softer stool. However, all fecal scores varied within a normal range [33]. Overall, many factors could influence the fecal consistency scores, such as undigested proteins and total dietary fiber content of plant protein sources as stated by Fricke [52] and Bednar et al. [33].

Fecal water content was not influenced by dietary treatments, and none of the dogs involved in the study showed any gastrointestinal disorders such as diarrhea. The fecal water content for both diets in our study varied by 70.0% and was similar to those previously reported in dogs fed either beef meat with plant protein or diets containing soy or added fiber [44, 50, 53]. However, Hill et al. [44] found that feces changed from very firm when 100% beef protein diet was fed, to soft when the high vegetable protein (soybean meal) diet was fed, and suggested that this was associated with an increase in fecal water content. Soluble fermentable fiber seems to cause a greater increase in fecal water than insoluble fiber (poorly fermented) as stated by Fahey et al. [50]. Nonetheless, this effect was not very pronounced; the feces maintained an adequate score without any loose stools detected during the current experiment. In contrast, Nery et al. [47] found a high water content in the feces of dogs fed poultry meal, and lower moisture in the feces of dogs fed diets containing wheat gluten. Protein digestion and absorption are considered to be one of the dietary factors affecting fecal water content [48]. However, the intake of diets containing large amounts of proteins, even if highly digestible, may exceed the digestive/absorptive capacity of the small intestine [54]. This may lead to a significant increase in protein reaching the hindgut available for proteolytic fermentation [51], consequently leading to greater water release into the intestinal lumen [48]. Thus, nutritional strategies to reduce fermentative activity in the colon by decreasing the quantity of undigested nutrients, such as proteins, should improve fecal quality [51]. Nevertheless, Nery et al. [21] found an increase in fecal water content, and negative effects on fecal quality in dogs receiving diets containing higher protein content (392 g crude protein/kg DM) compared to the dietary protein content used in the present study. It could be presumed that in the present trial, the vegetarian diet and meat-based diet did not affect fecal moisture because of their relatively moderate protein content (about 223 g/kg DM). If protein is present, but is not absorbed, the dietary amino acids in that protein are not available for the host, and provide nitrogen substrate for proteolytic bacteria, which may result in reduced stool quality [55].

## Conclusion

Consumer concerns about the adverse impacts of traditional meat-based diets are likely to increase due to connections between health conditions, farmed animal welfare concerns, environmental impacts and the provision of animal products accumulating. Accordingly, interest in alternative diets—including vegetarian diets—is likely to grow. It is absolutely possible for companion animals to survive, and indeed thrive, on vegetarian diets [2, 56–60] The data of this study appear to indicate that virtually the only significant difference between the meat-based diet and vegetarian diet was lower nitrogen-free extract digestibility in the vegetarian diet. However, there was no significant difference in amount of nitrogen excreted in feces or in the fecal scores.
